# Comparison of 5-aminolevulinic acid and MMP-14 targeted peptide probes in preclinical models of GBM

**DOI:** 10.7150/thno.107210

**Published:** 2025-02-24

**Authors:** Benjamin B. Kasten, Tingting Dai, Ke Jiang, Jennifer Coleman Clements, Kaixiang Zhou, Carlos A. Gallegos, Seth N. Lee, Anna G. Sorace, Hailey A. Houson, Logan D. Stone, James M. Markert, Jianghong Rao, Jason M. Warram

**Affiliations:** 1 Department of Otolaryngology, University of Alabama at Birmingham, Birmingham, AL 35294, USA.; 2 Departments of Radiology and Chemistry, Molecular Imaging Program at Stanford, Stanford University School of Medicine, Stanford, CA 94305, USA.; 3 Department of Neurosurgery, University of Alabama at Birmingham, Birmingham, AL 35294, USA.; 4 Department of Biomedical Engineering, University of Alabama at Birmingham, Birmingham, AL 35294, USA.; 5 Department of Radiology, University of Alabama at Birmingham, Birmingham, AL 35294, USA.; 6 O'Neal Comprehensive Cancer Center, University of Alabama at Birmingham, Birmingham, AL 35294, USA.

**Keywords:** MMP-14, GBM, NIRF, PET, 5-ALA

## Abstract

**Rationale**: Developing novel pre-operative and intraoperative imaging approaches for glioblastoma multiforme (GBM) could aid therapeutic intervention while sparing healthy normal brain, which remains a significant clinical challenge. 5-aminolevulinic acid (5-ALA) is the only intraoperative imaging agent approved to aid the resection of GBM. Matrix metalloproteinase 14 (MMP14), which is overexpressed in GBM, is an attractive target for preoperative and intraoperative imaging of GBM. Prior studies have shown the feasibility of near-infrared fluorescence (NIRF) imaging and positron emission tomography (PET) imaging of GBM xenografts in mice using MMP-14 targeted peptide probes. The present studies assessed the tumor-specific localization and contrast of these MMP-14 targeted peptides relative to 5-ALA in GBM models.

**Methods**: Fluorescence and PET imaging was performed after *i.v.* injection of 5-ALA and the MMP-14 targeted peptide probes (non-labeled or radiolabeled with ^64^Cu) in mice bearing human GBM orthotopic xenografts (U87, D54). Imaging signals were correlated to MMP-14 expression determined by immunofluorescence. Tumor-to-normal brain ratio (TBR) and Dice similarity coefficient (DSC) relative to tumor defined by *ex vivo* pathology or *in vivo* magnetic resonance imaging were determined for each imaging agent.

**Results:** NIRF signals from the MMP-14 targeted peptide probes showed comparable TBR (p < 0.05) but significantly higher DSC (p < 0.05) relative to 5-ALA. NIRF signals from the peptide probes significantly correlated with MMP-14 expression (p < 0.05). MMP-14 binding peptide labeled with ^64^Cu showed moderate DSC (0.45) while PET signals significantly correlated (p < 0.05) with NIRF signals from a co-injected MMP-14 substrate peptide. NIRF and PET signals localized in residual tumor regions in the resection cavity during *in situ* resection.

**Conclusions**: MMP-14 targeted peptides showed favorable TBR and higher tumor localization than 5-ALA in GBM orthotopic models. Further development of MMP-14 targeted peptide probes could lead to improved pre-operative and intraoperative management of GBM.

## Introduction

Glioblastoma multiforme (GBM) has the lowest rate of survival among primary brain malignancies in adults despite maximal safe surgical resection followed by radio-chemotherapy as the current standard of care 1. Neurosurgery remains a primary component of treatment, as patient survival correlates with a greater extent of resection of the tumor volume 1-3. Various preoperative and intraoperative approaches are used clinically to guide oncologic management of GBM 4-7, although 5-aminolevulinic acid (5-ALA) is currently the only intraoperative imaging agent approved by the United States Food and Drug Administration (FDA) to aid surgical resection. 5-ALA is converted into the fluorescent metabolite protoporphyrin IX (PpIX) in tumor cells, which produces favorable contrast to distinguish bulk GBM from adjacent normal brain. Intraoperative use of 5-ALA has been shown to increase rates of complete resection of contrast-enhancing GBM relative to pre-surgical MRI guidance alone, resulting in enhanced survival following resection 8-10. However, false positive signals and relatively low negative predictive values associated with 5-ALA 11-14 demonstrate a remaining clinical need for intraoperative imaging agents with greater specificity to distinguish between GBM invasion and normal brain or benign inflammatory regions beyond the bulk tumor. Alternative imaging agents intended for intraoperative surgical guidance of GBM are under preclinical and clinical investigation 15-17. Positron emission tomography (PET) imaging can also aid preoperative assessment of GBM by differentiating regions of tumor growth from benign pathologies that are difficult to distinguish through conventional anatomical imaging 7.

Overexpression of matrix metalloproteinase 14 (MMP-14) has been exploited for molecular imaging of GBM 18-20. MMP-14 expression in clinical tissues correlates with increased grade of malignancy and contributes to the diffuse pattern of GBM cell invasion into normal parenchyma 21-28. In previous work, our groups developed a set of peptide probes (substrate peptide, Figure S1; binding peptide, Figure S2; substrate-binding peptide, Figure S3) that specifically target MMP-14 for near-infrared fluorescence (NIRF) and PET imaging of GBM 29 (Figure S4). The “substrate peptide” (Figure S1) is initially non-fluorescent until the peptide sequence (RSCitG-HPhe-YLY 30-32) connecting the NIRF fluorophore (IRDye800) and quencher (IR QC-1) pair is cleaved by enzymatically active MMP-14 (Figure S4A). The “binding peptide” (Figure S2) containing a chelator (NOTA) and a peptide sequence (HWKHLHNTKTFL 33) that binds to the extracellular domain of MMP-14 is amenable for radiolabeling and PET imaging of MMP-14 expression (Figure S4B). Conjugation of these two peptides provides the “substrate-binding peptide” (Figure S3) that can be used for MMP-14 targeted NIRF and PET imaging (Figure S4C). Preclinical studies with human GBM cell lines and patient-derived xenograft orthotopic tumors *in vivo* showed favorable localization and signal contrast from these peptide probes in tumors relative to normal brain in mice 29.

The purpose of the present work was to further characterize the tumor-specific localization of the NIRF and PET signals from the MMP-14 targeted peptide probes indicated above relative to the performance of 5-ALA as a clinically validated intraoperative imaging agent for GBM. Quantitative correlates were used to compare imaging signals from the peptides, MMP-14 expression, and 5-ALA-induced PpIX fluorescence in brain tissues of mice containing human GBM orthotopic xenografts. Qualitative and quantitative imaging assessment was performed to determine if signals from the MMP-14 targeted peptide probes could reveal residual tumors during *in situ* resection of human GBM orthotopic xenografts. These studies provide a preclinical framework to evaluate multimodality targeted imaging approaches intended to guide surgical treatment of GBM.

## Materials and methods

### GBM xenograft implantation *in vivo*

Human GBM cell lines [D-54MG (D54) gifted from Darell D. Bigner, Duke University, Durham, NC; U-87MG (U87) obtained from the American Tissue Type Collection, Manassas, VA] were cultured as adherent monolayers in Dulbecco's Modified Eagle's Medium supplemented with 10% fetal bovine serum. 5-6 week old female athymic nude mice from Charles River (Wilmington, MA) were anesthetized and implanted with 500,000 GBM cells (5 µL suspension in serum-free medium) into the right caudate nucleus 2.5 mm lateral right to midline, 1 mm anterior to bregma and 3 mm depth over 2 minute infusion. Mice were monitored until fully recovered from anesthesia. Experiments were performed approximately 2 weeks (U87 xenografts) or 5 weeks (D54 xenografts) after implantation.

### *In vivo* MRI acquisition

Magnetic resonance imaging (MRI) of live mice was performed within 48 h of molecular imaging agents using a 9.4T preclinical MRI (Bruker BioSpin MRI GmbH, Ettlingen, Germany) equipped with an 86 mm inner-diameter volume coil for excitation and a 4-channel phased array brain surface coil for signal reception. Anesthetized mice had their heads immobilized using a bite bar and ear bars to reduce motion artifacts. High-resolution axial and coronal *T_2_*-weighted images of the brain were acquired using a multi-slice 2D rapid acquisition with relaxation enhancement (RARE) spin-echo pulse sequence. *T_2_* RARE scan parameters were as follows: TR/TE = 2500/40 ms, NEX = 6, RARE factor = 8, FOV = 20 x 20 mm^2^, acquisition matrix = 256 x 256, and slice thickness = 0.5 mm. For comparison with PET/CT images, MRI axial images were resampled using VivoQuant software (version 2022, Invicro).

### Dosing of imaging agents

All solutions used for dosing were brought to a final volume of 100-200 µL and administered *i.v.* via tail vein. 5-ALA was administered at 0.1 mg/g body weight to the cohorts indicated below. Unless noted otherwise, mice were anesthetized and euthanized 4-5 h after dosing.

*Dosing of substrate peptide and 5-ALA*: tumor-bearing mice (D54, n = 4; U87, n = 5) received a single solution in PBS containing approximately 3 nmol substrate peptide mixed with 5-ALA.

*Dosing of substrate-binding peptide and 5-ALA*: tumor-bearing mice (U87, n = 5) received separate solutions of 5-ALA and approximately 3 nmol substrate-binding peptide dissolved in PBS containing 5% ethanol; injections of 5-ALA and the peptide were separated by 30 min.

*Dosing of cocktail mixture of [^64^Cu]Cu-binding peptide, substrate peptide, and 5-ALA*: tumor-bearing mice (D54, n = 4; U87, n = 2) received a solution in PBS containing approximately 1.1 nmol [^64^Cu]Cu-binding peptide (5.3 MBq), 2.7 nmol substrate peptide, and 5-ALA.

*Dosing of [^64^Cu]Cu-substrate-binding peptide*: tumor-bearing mice (U87, n = 2) received approximately 0.4 nmol [^64^Cu]Cu-substrate-binding peptide (2.5 MBq) in PBS containing 5% ethanol.

### *Ex vivo* NIRF imaging

Cohorts of mice were anesthetized and euthanized 4-5 h *p.i.* of the non-labeled peptides. The skin over the skull was removed and NIRF signal in the head and brain was imaged on a Pearl system (LI-COR) prior to and following removal of the skull cap. In sub-sets of mice, in-situ surgical resection of macroscopically visible tumor was performed with additional acquisition of mid-resection and post-resection NIRF images. Resected tissues and residual brains were fixed in 10% formalin overnight for subsequent processing. For all remaining mice, the whole brains were resected, fixed in 10% formalin overnight, and imaged by the Pearl system. Brains and resected tissues were then serially sectioned (1 mm coronal slices), and all slices were imaged by the Pearl system for quantitative NIRF assessment. Three or four slices per mouse containing tumor and adjacent normal brain without tumor were then imaged on an Odyssey CLx NIRF scanner (LI-COR) for qualitative NIRF “high resolution” assessment. Each tissue slice was then imaged as described below for PpIX fluorescence. One representative tumor-bearing slice per mouse was dehydrated in 70% EtOH, processed for embedding in paraffin, and further sectioned (5 µm) for H+E staining and for additional unstained formalin fixed paraffin embedded (FFPE) sections. Unstained FFPE sections were imaged on an Odyssey scanner (LI-COR). All NIRF images were analyzed using ImageStudio software (V3.1.4, LI-COR). NIRF “low resolution” signals from the Pearl system were used to determine mean fluorescence intensity (MFI) values of the tumor area and contralateral normal brain by drawing manual regions of interest (ROIs) based on grossly visible tumor in 1 mm brain slices.

### *Ex vivo* PpIX fluorescence imaging

PpIX fluorescence resulting from 5-ALA was imaged *ex vivo* in 1 mm formalin-fixed brain slices on a stereomicroscope as previously described 34. Briefly, tissues were illuminated using an external ultraviolet (405 nm) LED source while fluorescence was acquired using a custom Leica, MZFLIII Stereo Microscope (Leica). A liquid-crystal tunable wavelength filter in the Leica Spectral Camera was set for collection of emission images from 600 to 720 nm in 10-nm increments, each acquired for 5 sec. Composite fluorescence images (unmixed composites) were generated for each image cube by separating the spectral signature of PpIX from those of background autofluorescence using a spectral library that was compiled from numerous spectral profiles collected from control tissue. A corresponding brightfield image of each brain slice was also collected using the stereomicroscope during white light (brightfield) illumination. Mean fluorescence intensity (MFI) values of the tumor area and contralateral normal brain were quantified using ImageJ (v1.53) by drawing manual ROIs based on grossly visible tumor in 1 mm brain slices and H+E stained tissue sections obtained from the same brain slice.

### Spatial correlation analysis of NIRF, PpIX and H+E

The 2-dimensional image processing workflow is diagrammed in Figure S5. Raw images of *ex vivo* NIRF (low resolution quantitative signals) and PpIX fluorescence signals in 1 mm formalin-fixed brain slices and from corresponding H+E stained tissue sections were used to generate tissue masks and region masks (signal-intense area in fluorescence images, tumor area in brightfield and H+E images). Processed mask images were resampled and registered using MATLAB (R2022b, Mathworks, Inc.) using custom code (available upon request). DSC was calculated 35 based on 2-dimensional area for each fluorescence imaging agent relative to tumor pixel area defined by H+E.

### *In vivo* PET/CT imaging

*A*t 2.5-4 h p.i. of the [^64^Cu]Cu-labeled peptide solutions, live mice were imaged by PET/CT using a GNEXT small animal scanner (Sofie Biosciences, Culver City, CA) with a PET energy window of 350-650 KeV (15 min acquisition) and CT voltage of 80 kVp (current 150 μA, 720 projections). The PET images were reconstructed using a 3-Dimensional-Ordered Subset Expectation Maximization algorithm (24 subsets and 3 iterations), with random, attenuation, and decay correction*.* The CT images were reconstructed using a Modified Feldkamp Algorithm. Reconstructed images were analyzed using VivoQuant software. Following imaging, anesthetized mice were euthanized and whole brains were resected, weighed, fixed in 10% formalin overnight, serially sectioned (1 mm coronal slices), and processed for further NIRF imaging, paraffin embedding, and tissue sectioning for H+E or immunofluorescence analyses as described below. Alternatively, in sub-sets of mice, the heads were isolated from the carcass of euthanized mice, the skull cap was removed, and in-situ surgical resection of the tumor was performed with additional PET/CT acquisitions when part of the macroscopic tumor was resected (mid-resection PET) and after complete resection of the macroscopically visible tumor (post-resection PET).

### Co-registration and multimodality image analysis of PET and MRI

The 3-dimensional image processing workflow is diagrammed in Figure S6. The Brain Atlas automated tool of VivoQuant was used to segment brain regions from the reconstructed PET/CT and MRI, with manual adjustment of the initially segmented volume as needed to ensure the entire brain was included. The resulting PET/CT brain scans were automatically resampled and registered to the MRI using a rigid registration approach (Reorientation/Registration tool, VivoQuant). Tumor (*T_2_*-weighted MRI enhancing tumor region) and normal brain VOIs were manually drawn on the MRI segmented brain images and copied to the fused PET segmented images for analyses of mean standardized uptake value (SUV_mean_) and maximum standardized uptake value (SUV_max_). For determination of DSC between PET and MRI, iso-contour VOIs corresponding to different thresholds (20% to 80%) of the tumor SUVmax were generated using the “global threshold” function within VivoQuant; the resulting PET and MRI DICOM files of the segmented VOIs (region mask) were analyzed in MATLAB (R2022b, Mathworks, Inc.). Volumetric DSC was calculated as previously described 35, 36 for each PET imaging agent relative to MRI-defined tumor volume.

### Statistical analyses

Data were analyzed using Microsoft Excel or GraphPad Prism (Version 10.0.2, GraphPad Software, La Jolla, CA, USA). Unless indicated otherwise, individual data points shown in graphical data indicate values for each mouse in the respective group. Statistical outliers were identified using the ROUT function (Q = 2%) in GraphPad Prism and were excluded from subsequent analyses. Paired or unpaired *t*-tests as appropriate were used when comparing two groups. When comparing multiple groups, one-way ANOVA tests, followed by Dunn's multiple comparisons test where appropriate. All *p*-values correspond to two-tailed tests; significance was considered to be at *p* < 0.05.

## Results

### MMP-14 targeted peptide probes show localized NIRF signal in bulk tumor and microscopic deposits of human GBM orthotopic xenografts in nude mice

**Figure 1:**
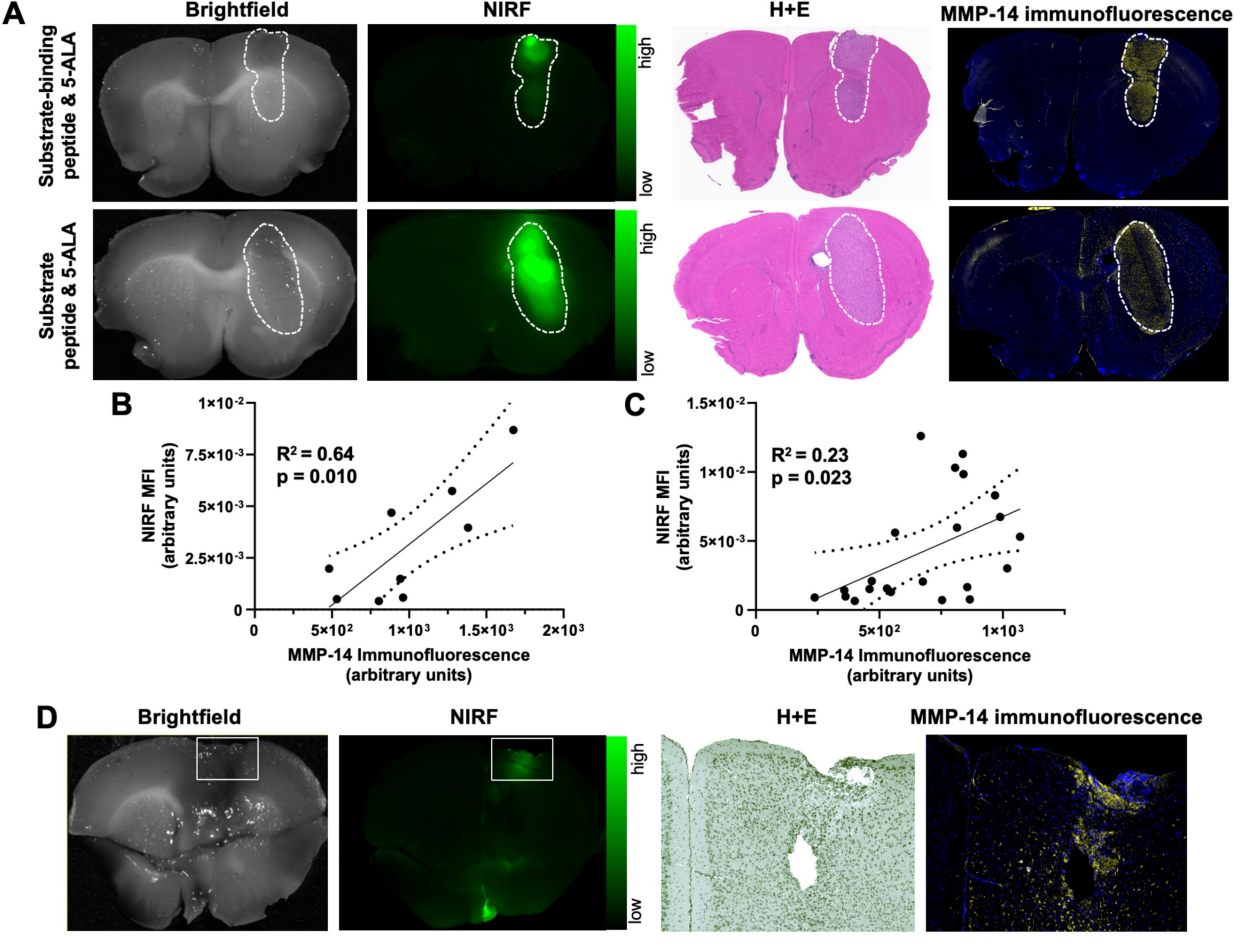
NIRF signal from MMP-14 targeted peptide probes localizes in macroscopic and microscopic GBM orthotopic xenografts and correlates with histological MMP-14 expression. A, representative images of fixed brain slices from GBM-bearing mice dosed *i.v.* with the substrate-binding peptide and 5-ALA (top panels) or the substrate peptide and 5-ALA (bottom panels); corresponding brightfield and NIRF panels are from the same 1 mm brain slice, which was also used to obtain histologic sections for the corresponding H+E and MMP-14 immunofluorescence (yellow; blue, nuclei stained by DAPI) panels. White dotted line indicates tumor region. B-C, least-squares fit of linear regression between histological MMP-14 immunofluorescence MFI and NIRF signal MFI in 1 mm brain slices from GBM-bearing mice dosed *i.v.* with the substrate-binding peptide (B) or the substrate peptide (C); dotted lines show the 95% confidence interval of the correlation. D, brightfield and NIRF images of a 1 mm fixed brain slice from a mouse dosed *i.v.* with the substrate peptide, with white box indicating the region of implantation and the corresponding zoomed in areas from histology sections stained with H+E or MMP-14 immunofluorescence (4x original magnification). MFI, mean fluorescence intensity; a.u., arbitrary units.

The present studies first determined the NIRF contrast from the MMP-14 targeted peptide probes in multiple human GBM orthotopic xenograft models that express MMP-14, as a continuation of our prior work using these probes in a single orthotopic model of GBM 29. Groups of mice bearing U87 or D54 orthotopic xenografts were administered (*i.v.*) 5-ALA in combination with either the substrate peptide or the substrate-binding peptide and euthanized 4 h later. NIRF imaging of formalin-fixed 1 mm slices from resected brains of these mice indicated each peptide probe specifically localized within bulk tumor regions (Figure 1A), while significantly lower signal was apparent in adjacent and contralateral normal brain areas (p < 0.01, p < 0.05 for substrate peptide and substrate-binding peptide, respectively; Table 1). Immunofluorescence staining of brain sections from these mice showed significantly greater expression of MMP-14 in the orthotopic xenografts relative to normal brain (p < 0.0001, Figure S7A-B), as anticipated based on results from alternative GBM models 19, 29, 32. While prior *in vitro* immunocytochemistry analyses indicated higher expression of MMP-14 in U87 cells relative to D54 cells 29, orthotopic U87 and D54 xenograft tumors in the present studies did not show a significant difference in the tumor-to-normal brain ratio (TBR) for MMP-14 immunofluorescence (p>0.05, Figure S7C). This indicated that both human GBM orthotopic xenograft models were suitable for MMP-14 targeted imaging assessments with the peptide probes. NIRF signal intensities in the bulk tumor and contralateral normal brain regions correlated with the corresponding MMP-14 immunofluorescence signal observed in U87 and D54 orthotopic xenografts (Figure 1B-C). This correlation was stronger for the substrate-binding peptide (R^2^ = 0.64, Figure 1B) than for the substrate peptide (R^2^ = 0.23, Figure 1C). Surprisingly, one of the mice in the U87 xenograft cohort did not exhibit macroscopic tumor growth at the time the studies were performed, although NIRF signal was observed in the brain of this mouse where implantation occurred. Histological analysis of this brain section indicated microscopic growth of the GBM xenograft (Figure 1D), which confirmed the tumor-specific NIRF signal observed in this region.

### 5-ALA and MMP-14 targeted peptide probes produce similar contrast in human GBM orthotopic xenograft bulk tumor relative to contralateral normal brain in nude mice

**Figure 2:**
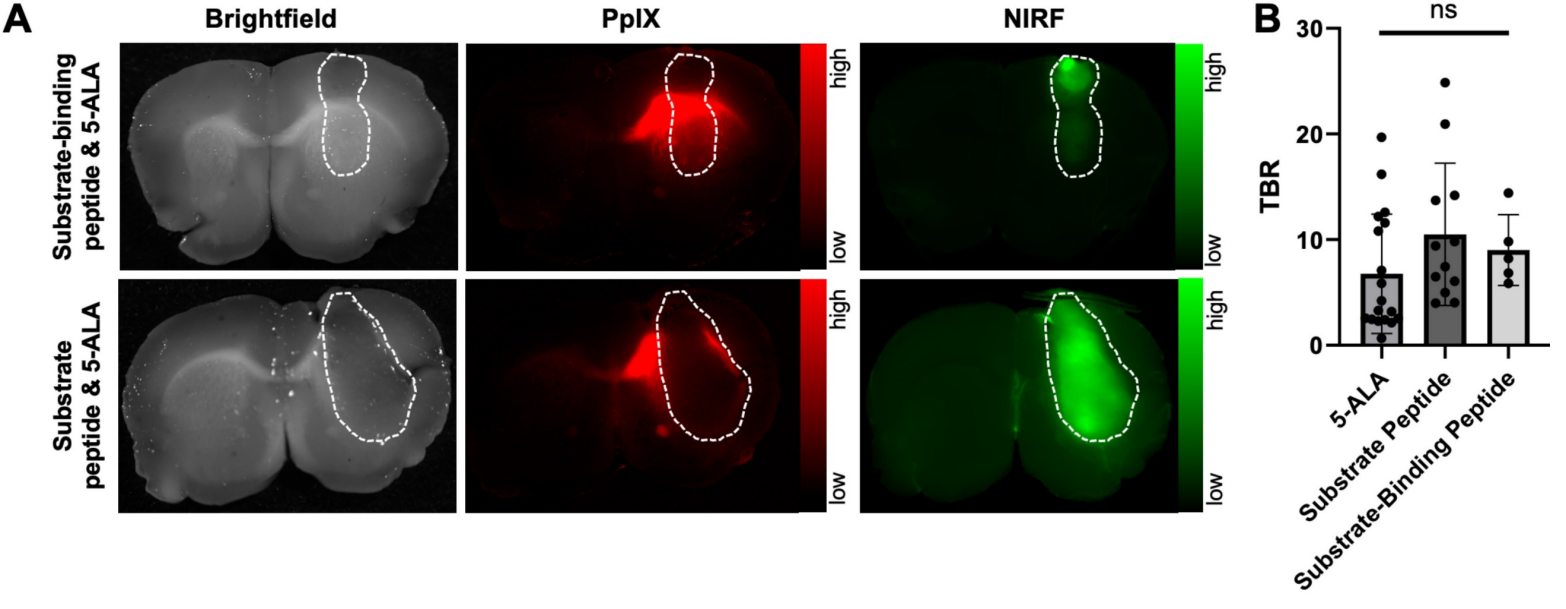
PpIX fluorescence from 5-ALA and NIRF from MMP-14 targeted peptide probes provide comparable tumor-to-normal brain ratios in GBM orthotopic xenografts. A, representative images (brightfield, PpIX fluorescence, NIRF) of 1 mm fixed brain slices from GBM-bearing mice dosed *i.v.* with 5-ALA and the substrate-binding peptide (top panels) or 5-ALA and the substrate peptide (bottom panels); corresponding panels are from the same 1 mm brain slice. White dotted line indicates tumor region. B, mean ± SD tumor-to-normal brain ratio (TBR) of mean fluorescence intensity for each imaging agent quantified in 1 mm fixed brain slices; significance determined by ANOVA (ns, not significant).

A second goal of these studies was to compare the fluorescence contrast in GBM models relative to normal brain when using 5-ALA or the MMP-14 targeted peptide probes as imaging agents. The distinct fluorescence excitation and emission spectral profiles between PpIX (λ_ex_= 405 nm, λ_em_= 635 nm) and IRDye800 (λ_ex_ = 773 nm, λ_em_ = 792 nm) enabled direct comparison of PpIX fluorescence from 5-ALA and NIRF from the peptide probes within the same brain tissue slice from the studies above. Similar to the NIRF contrast observed from the MMP-14 targeted peptide probes, PpIX fluorescence (Figure 2A) was significantly higher in bulk GBM xenografts relative to contralateral normal brain regions (p < 0.001, Table 1) of mice. PpIX fluorescence in the bulk tumor and contralateral normal brain weakly correlated with MMP-14 immunofluorescence signals in corresponding brain regions (R^2^ = 0.29, Figure S8). Fluorescence TBRs (Figure 2B) resulting from 5-ALA, the substrate peptide, and the substrate-binding peptide (6.8 ± 5.7, 10.5 ± 6.7, 9.0 ± 3.4, respectively) across mice were not significantly different (p>0.05). These results confirmed that the peptide probes successfully delineated human GBM orthotopic xenografts from normal brain with similarly high contrast as 5-ALA.

### Spatial overlap between fluorescence and tumor is significantly greater with MMP-14 targeted peptide probes than 5-ALA in human GBM orthotopic xenografts in nude mice

**Figure 3:**
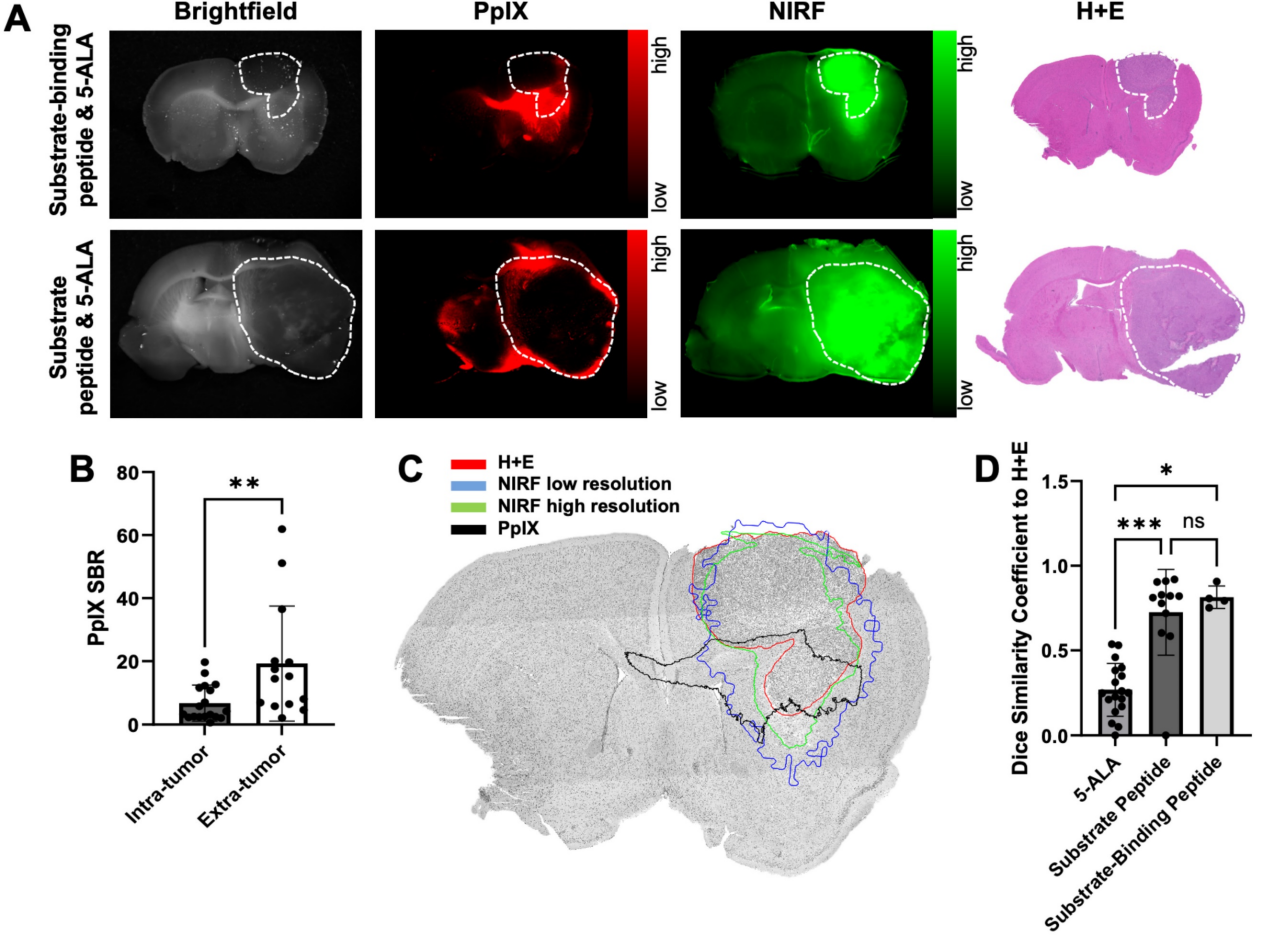
NIRF from MMP-14 targeted peptide probes exhibit significantly greater tumor localization relative to PpIX fluorescence from 5-ALA in GBM orthotopic xenografts. A, representative images of fixed brain slices from GBM-bearing mice dosed *i.v.* with 5-ALA and the substrate-binding peptide (top panels, U87 xenograft) or 5-ALA and the substrate peptide (bottom panels, D54 xenograft); corresponding brightfield, PpIX, and NIRF panels are from the same 1 mm brain slice, which was also used to obtain a histologic section for the corresponding H+E panel. White dotted line indicates tumor region. B, mean ± SD signal-to-normal brain ratio (SBR) of PpIX fluorescence quantified in intra-tumor and extra-tumor regions of fixed brain slices from GBM-bearing mice (significance determined by unpaired *t*-test; **p < 0.01). C, representative H+E image of a brain section from a U87 GBM-bearing mouse dosed *i.v.* with 5-ALA and the substrate-binding peptide; overlaid lines indicate registered regions of tumor (red, H+E) or fluorescence signals (blue, NIRF low resolution Pearl system; green, NIRF high resolution Odyssey scanner; black, PpIX fluorescence) determined in the 1 mm fixed brain slice from which the histology section was obtained. D, mean ± SD 2-dimensional Dice similarity coefficient of each imaging agent relative to tumor region defined by H+E in fixed brain slices from GBM-bearing mice (significance determined by ANOVA followed by Dunn's multiple comparisons test; *, p < 0.05; ***, p < 0.001; ns, not significant).

Closer inspection of PpIX fluorescence signals in the GBM-bearing brain tissue slices revealed notable fluorescence contrast outside of the gross tumor boundary (Figure 2A, Figure 3A), similar to results observed in prior work using 5-ALA in nude mice with GBM orthotopic xenografts 34, 37, 38. Extra-tumor contrast was apparent in proximal normal brain regions, notably white matter tracts (e.g., corpus callosum) in the ipsilateral hemisphere, as well regions distal to xenograft growth. Quantification of PpIX fluorescence signal contrast relative to contralateral normal brain (signal-to-normal brain ratio, SBR) in these extra-tumor regions indicated a significantly greater SBR (19.3 ± 18.2) than measured within intra-tumor regions (6.8 ± 5.7, p < 0.01; Figure 3B). The area of extra-tumor PpIX fluorescence relative to all fluorescence-positive area was higher in gross brain slices from mice bearing U87 xenografts (83 ± 12%) relative to those bearing D54 xenografts (39 ± 28%, Figure S9A-B). This was likely due to the relative difference in xenograft size between the two GBM models at the time of the studies, as D54 tumors took up a larger percentage of the brain section area (27 ± 12%) than did the U87 tumors (12 ± 8%) (Figure S9B). There was a weak inverse correlation between percent tumor area within the brain section and the percent of extra-tumor PpIX fluorescence area across both xenograft models in these studies (Figure S9C). Sparse regions of intra-tumoral necrosis were visible in H+E stained sections of several D54 GBM xenografts (Figure 3A), but not in most U87 GBM xenografts (Figure 2A, Figure 3A) in the present studies. Interestingly, PpIX TBRs were not significantly different between GBM xenografts with or without necrosis (4.3 ± 4.2 and 8.0 ± 6.0, respectively, p>0.05). While intra-tumor PpIX signals throughout GBM tumor regions were qualitatively and quantitatively lower than in extra-tumor regions, NIRF contrast from the MMP-14 targeted peptide probes remained strong throughout the GBM tumor region, including regions showing necrosis in H+E stained sections (Figure 3A).

Low extra-tumor NIRF signals from the MMP-14 targeted peptide probes were observed from gross brain slices of mice bearing either U87 or D54 GBM xenografts (Figure 2A, Figure 3A). NIRF imaging displayed notable contrast throughout the grossly visible and pathologic tumor regions. A representative image showing overlaid areas for PpIX fluorescence, NIRF from the substrate-binding peptide, and H+E defined tumor is shown in Figure 3C. The Dice similarity coefficient (DSC) for 5-ALA (0.27 ± 0.16, Figure 3D), determined by the PpIX fluorescence pixel area and the tumor pixel area defined by pathology, was significantly lower (p < 0.05) than the DSC for the NIRF pixel area corresponding to the substrate peptide or the substrate-binding peptide relative to the tumor pixel area (0.73 ± 0.25 and 0.81 ± 0.07, respectively, Figure 3D). DSC values were not significantly different between the peptide probes (p>0.05). These results indicate relatively high spatial overlap between the activated NIRF signal from the peptide probes and GBM orthotopic xenografts relative to the PpIX fluorescence signal produced after administering 5-ALA to mice.

### A cocktail of MMP-14 targeted peptide probes allows GBM-specific dual-modality PET and NIRF imaging of human GBM orthotopic xenografts in nude mice

**Figure 4:**
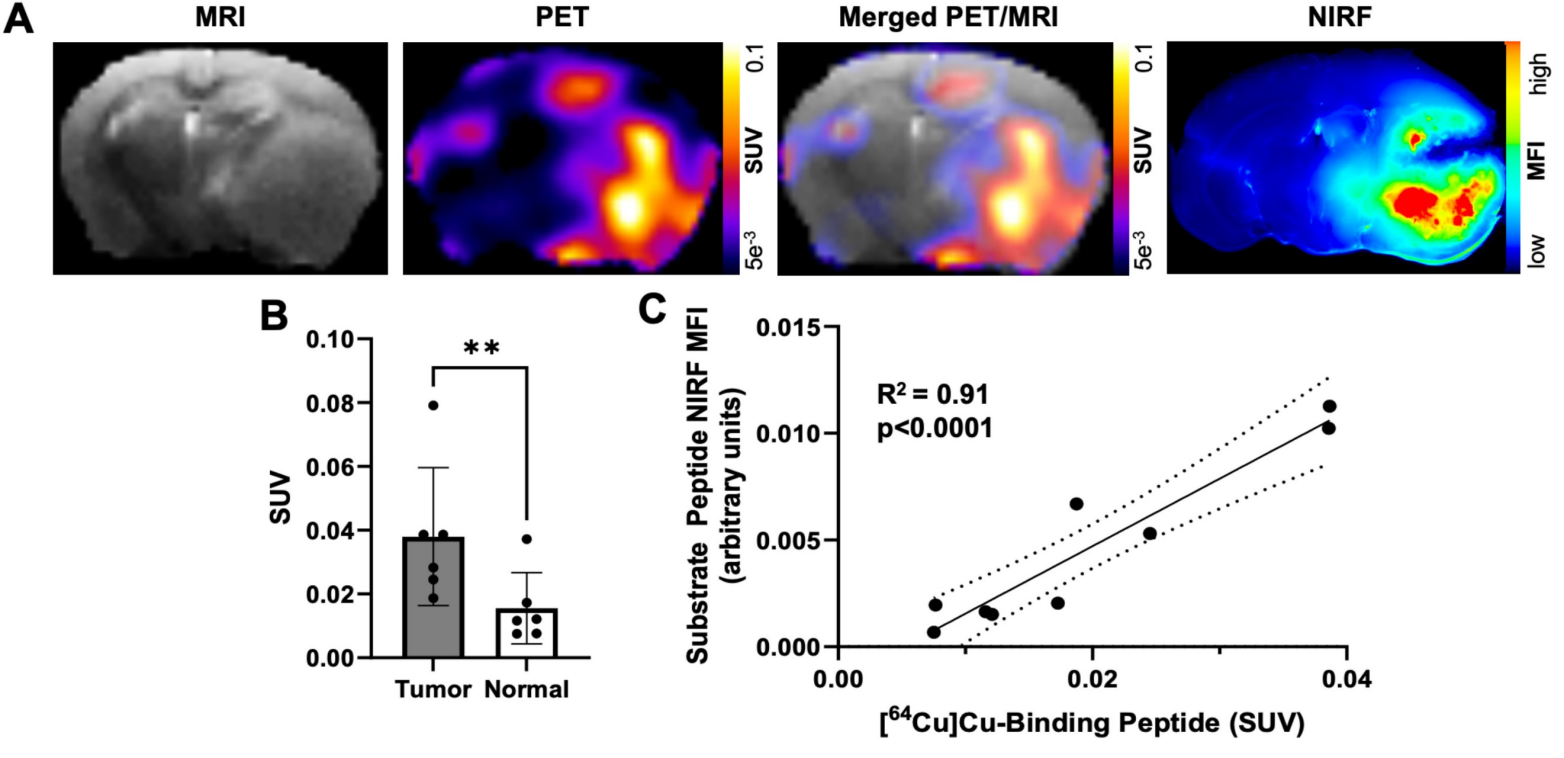
A cocktail of MMP-14 targeted peptide probes results in tumor localization and correlation of PET and NIRF signals in GBM orthotopic xenografts. A, representative images of segmented, registered brain regions from *in vivo T_2_*-weighted MRI and PET imaging of a GBM-bearing mouse dosed *i.v.* with [^64^Cu]Cu-binding peptide, substrate peptide, and 5-ALA; NIRF panel is from a 1 mm fixed brain slice imaged *ex vivo*. B, mean ± SD SUV_mean_ in tumor and contralateral normal brain regions of mice dosed *i.v.* with [^64^Cu]Cu-binding peptide; significance determined by paired *t-*test (**, p < 0.01). C, least-squares fit of linear regression between *in vivo* [^64^Cu]Cu-binding peptide SUV_mean_ and *ex vivo* substrate peptide NIRF MFI in fixed brain slices; dotted lines show the 95% confidence interval of the correlation. a.u., arbitrary units.

Our previous studies with the binding peptide utilized ^68^Ga for PET imaging and biodistribution 29. In the present studies, ^64^Cu was utilized as a longer-lived radionuclide to enable *in vivo* PET imaging of GBM orthotopic xenografts at 4 h post-injection of the peptide to match the time point of the *ex vivo* NIRF imaging studies. The binding peptide showed near quantitative complexation of ^64^Cu (43.5-76.6 MBq) at room temperature within 20 min to produce the desired radiolabeled product ([^64^Cu]Cu-binding peptide; Figure S10). The substrate peptide and 5-ALA were added to this crude reaction solution of [^64^Cu]Cu-binding peptide (5.2 GBq/µmol molar activity at time of dosing) to generate a cocktail mixture of the imaging agents, which was administered *i.v.* to mice bearing D54 or U87 GBM orthotopic xenografts. PET imaging of live mice 4 h after dosing qualitatively showed similar spatial distribution from uptake of activity to the anatomical tumor region indicated by MRI; representative PET and MRI images from segmented brain regions are shown in Figure 4A. A significantly higher SUV_mean_ was observed within tumor regions defined by *T_2_*-weighted MRI relative to contralateral normal brain (0.038 ± 0.022 and 0.016 ± 0.011, respectively; p < 0.01, Figure 4B). The observed standardized uptake value ratio (SUVR: ratio of tumor SUV_mean_ to normal brain SUV_mean_) was 2.6 ± 0.7 across the mice in these studies. A significant correlation was not observed between the *in vivo* [^64^Cu]Cu-binding peptide PET SUV_mean_ in the *T_2_*-weighted MRI tumor volume and the *ex vivo* MMP-14 immunofluorescence signal (MFI) in resected brain sections (Figure S11).

*Ex vivo* imaging of formalin-fixed 1 mm brain slices from these mice showed anticipated localization and spatial distribution of the activated NIRF signal from the substrate peptide within the tumor (Figure 4A). A strong correlation was determined between the *in vivo* [^64^Cu]Cu-binding peptide PET SUV_mean_ signal and the *ex vivo* substrate peptide NIRF MFI signal within corresponding brain regions (R^2^ = 0.91, p < 0.001; Figure 4C), indicating the utility of the cocktail dosing strategy containing the separate MMP-14 targeted PET and NIRF peptide probes for dual-modality imaging in these GBM orthotopic xenografts (Figure S4D).

### Radiolabeled MMP-14 targeted peptide probes show moderate overlap with MRI-defined regions of human GBM orthotopic xenografts in nude mice

**Figure 5:**
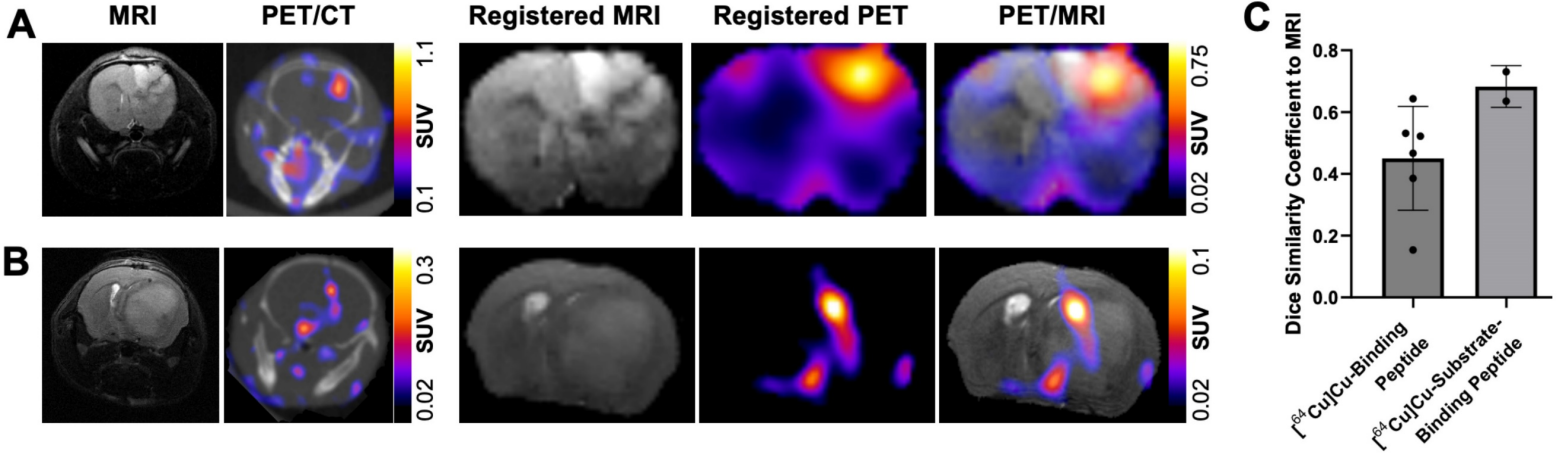
PET signals from radiolabeled MMP-14 targeted peptide probes spatially localize within MRI-defined tumor regions in GBM orthotopic xenografts. A-B, representative images of *in vivo T_2_*-weighted MRI, PET/CT, and segmented, registered brain regions from MRI and PET images of GBM-bearing mice dosed *i.v.* with [^64^Cu]Cu-substrate-binding peptide (A) or [^64^Cu]Cu-binding peptide (B). C, mean ± SD 3-dimensional Dice similarity coefficient of each radiolabeled peptide relative to tumor region defined by *T_2_*-weighted MRI.

*In vivo* MRI assessment of tumor-bearing mice in the present studies enabled volume-wise spatial analyses between the GBM orthotopic xenografts and the radiolabeled MMP-14 targeted peptide probes. As shown in Figure 5A, comparison between MRI, PET/CT and registered segmented brain images of U87 GBM orthotopic xenografts in mice administered [^64^Cu]Cu-substrate-binding peptide demonstrated localization of the PET signal within the MRI-defined tumor region in the current studies. Similar comparison of PET/CT, MRI, and registered segmented brain images from GBM-bearing mice that received the [^64^Cu]Cu-binding peptide qualitatively confirmed the tumor-specific localization of the PET signal (Figure 5B). DSC analyses were performed to evaluate the voxel-wise spatial overlap throughout the brain for the PET peptide probes at various thresholds of the observed SUV_max_ within the tumor relative to the *T_2_*-weighted MRI enhancing tumor region. These quantitative spatial analyses indicated a DSC of 0.68 ± 0.07 at a 30% threshold of the tumor SUV_max_ for the [^64^Cu]Cu-substrate-binding peptide and a DSC of 0.45 ± 0.17 at a 20% threshold of the tumor SUV_max_ for the [^64^Cu]Cu-binding peptide (Figure 5C). At these thresholds, 70.3 ± 9.5% and 44.5 ± 23.3% of the positive PET voxels from the [^64^Cu]Cu-substrate-binding peptide and [^64^Cu]Cu-binding peptide, respectively, were within the tumor. These PET signals covered 66.5 ± 4.3% and 58.6 ± 16.9%, respectively, of the MRI-defined tumor volume. In summary, PET imaging with the radiolabeled MMP-14 targeted peptide probes showed moderate spatial overlap with the *T_2_*-weighted MRI volume of GBM orthotopic xenografts in these studies.

### Mid-resection *in situ* imaging shows NIRF and PET signals in resected and residual tumor regions of human GBM orthotopic xenografts in nude mice

**Figure 6:**
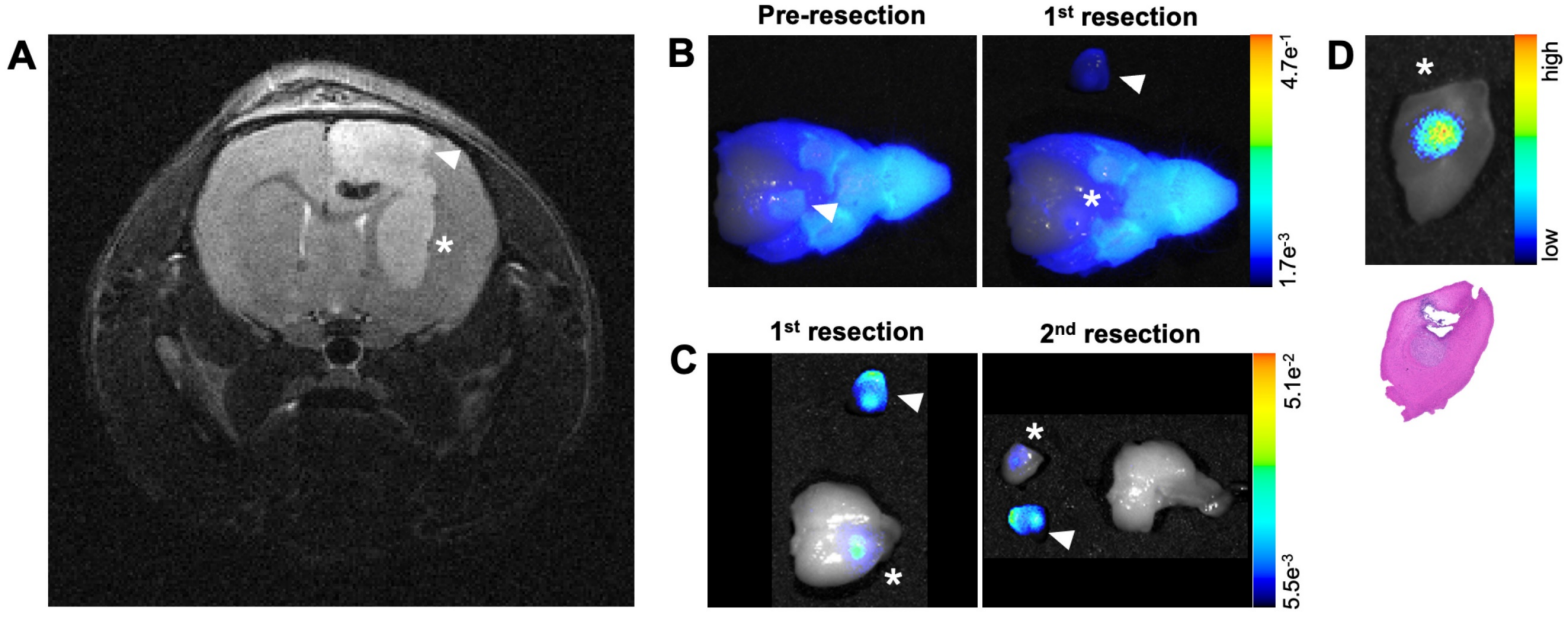
MMP-14 substrate-binding peptide shows NIRF signal in residual tumor during mock resection of a GBM orthotopic xenograft. A, pre-resection *in vivo T_2_*-weighted MRI of a of GBM-bearing mouse used for mock resection. B, NIRF/brightfield overlay images of the GBM-bearing mouse head during *in situ* resection after opening the skull cap prior to removing tumor (Pre-resection) and after resection of the superior xenograft portion (1^st^ resection). C, NIRF/brightfield overlay images of the extracted whole brain with resected xenograft portions after 1^st^ and 2^nd^ resections of xenograft portions containing NIRF signal. D, NIRF/brightfield overlay image of fixed tissue (top panel) and H+E stained section indicating pathological tumor (bottom panel) from the 2^nd^ resection containing the inferior portion of the GBM orthotopic xenograft. Arrowhead and asterisk across panels indicate superior and inferior portions, respectively, of the GBM orthotopic xenograft.

Another goal of these studies was to determine if imaging signals from the MMP-14 targeted peptide probes could reveal residual GBM in the resection cavity during a debulking surgical procedure. *In situ* NIRF imaging during exploratory tumor resection was conducted on a mouse from the U87 GBM cohort that was administered the non-labeled substrate-binding peptide. MRI prior to mock resection (Figure 6A) indicated multi-lobed growth of the GBM xenograft. *In situ* NIRF imaging of the brain after removing the skull cap showed NIRF signal in the grossly visible, superior bulk tumor region pre-resection (Figure 6B) and in non-resected tumor in the inferior portion of the resection cavity after the 1^st^ resection (Figure 6B). NIRF contrast was clearly visible in this inferior tumor portion after extracting the brain (Figure 6C), when the inferior tumor portion was further resected (Figure 6C) including a margin of normal-appearing brain tissue for more thorough investigation of the localized NIRF signal post-resection. NIRF imaging and histological analysis confirmed the presence of GBM with tumor-specific localization of the NIRF signal relative to surrounding normal brain in the inferior resected specimen (Figure 6D).

**Figure 7:**
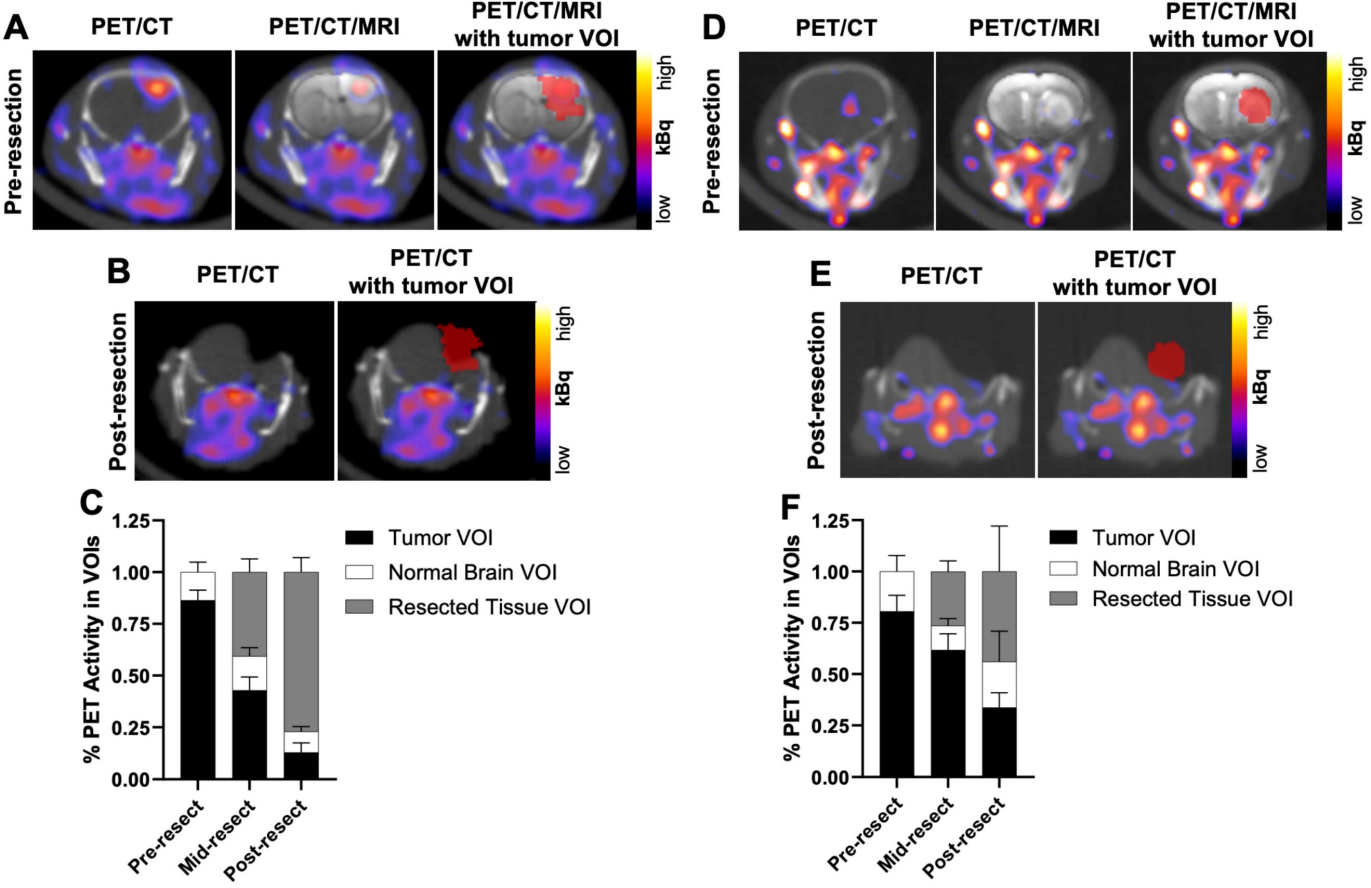
Mid-resection PET signals from radiolabeled MMP-14 targeted peptide probes were consistent with resected and residual intracranial tumor regions of GBM orthotopic xenografts. A, D, representative *in vivo* PET/CT, PET/CT with overlaid *T_2_*-weighted MRI segmented brain region (PET/CT/MRI), and PET/CT/MRI with overlaid tumor VOI (red) pre-resection images from GBM-bearing mice dosed *i.v.* with [^64^Cu]Cu-substrate-binding peptide (A) or [^64^Cu]Cu-binding peptide (D). B, E, representative *in situ* PET/CT and PET/CT with overlaid tumor VOI (red) post-resection images from GBM-bearing mice dosed *i.v.* with [^64^Cu]Cu-substrate-binding peptide (B) or [^64^Cu]Cu-binding peptide (E). C, F, mean ± SD percent PET activity in intracranial tumor VOI, contralateral normal brain VOI, and resected tissue VOI at respective pre-resection, mid-resection, and post-resection *in situ* PET/CT imaging sessions of GBM-bearing mice dosed *i.v.* with [^64^Cu]Cu-substrate-binding peptide (C, *n* = 2 mice/group) or [^64^Cu]Cu-binding peptide (F, *n* = 2 mice/group). VOI, volume of interest.

To determine if PET signals could similarly detect residual GBM during resection, U87 orthotopic xenograft-bearing mice (n = 2) underwent *in situ* resection 3 to 5 h *p.i.* the [^64^Cu]Cu-substrate-binding peptide. Figure 7A presents representative merged PET/CT/MRI pre-resection images indicating the location of the tumor volume of interest (VOI) for reference of activity throughout the course of resection. After opening the skull and resecting a portion of the grossly visible tumor, a mid-resection PET/CT scan indicated approximately 43% of the [^64^Cu]Cu-substrate-binding peptide activity among all VOIs (tumor, adjacent normal brain, resected tissue) remained in the pre-resection tumor VOI (Figure 7C). Additional resection of tissue followed by a post-resection PET/CT scan (Figure 7B) showed 13% of the activity among all VOIs was present in the tumor VOI (original pre-resection tumor volume), while 77% of the activity was located in the resected tissue VOIs (Figure 7C). Similar *in situ* resection experiments using a cocktail of the substrate peptide and the [^64^Cu]Cu-binding peptide were performed in mice bearing D54 orthotopic xenografts (n = 2 mice). The majority of the PET signal in the tumor VOI prior to resection (Figure 7D) was observed in the resected tissue components as indicated in mid- and post-resection (Figure 7E) PET/CT scans. These analyses revealed 62% and 34%, respectively, of the [^64^Cu]Cu-binding peptide activity among all VOIs remained in the tumor VOI region during the mid- and post-resection PET/CT scans (Figure 7F), while resected tissue VOIs contained 26% and 44%, respectively, of the activity at each time point.

The collective results from these preliminary NIRF and PET *in situ* resection experiments support the conclusion that mid-resection imaging with the MMP-14 targeted peptide probes enables detection of partially resected GBM xenograft regions remaining in the surgical cavity.

## Discussion

The goal of molecular image-guided surgery is to maximize the extent of safe surgical resection, which directly impacts the prognosis of GBM. Enzymatically active MMP-14 is significantly overexpressed in GBM, including the xenograft models (U87, D54) utilized in the present studies, while expression is low in human and murine normal cerebral tissue 19, 21, 24. Thus, MMP-14 is an attractive molecular target for novel imaging agents designed to aid preoperative or intraoperative assessment of GBM. The observed direct correlation between MMP-14 expression and the activated NIRF signal from the MMP-14 targeted peptide probes is consistent with target-mediated localization of the peptides in these human GBM orthotopic xenografts. Importantly, there was clear spatial overlap of signaling, although there was not a significant correlation between MMP-14 expression and the PET signal from the [^64^Cu]Cu-binding peptide. This was likely due to confounding effects when comparing *in vivo* 3-dimensional volumetric data to *ex vivo* 2-dimensional immunofluorescence from tissue sections, which may not be representative of the entire tumor volume. Developing approaches that quantify 3-dimensional tumor heterogeneity of molecular expression would aid evaluation of the *in vivo* performance of imaging probes.

The observed differences in spatial localization between the peptide probes were anticipated due to the differing structure of each peptide probe. The presence of the MMP-14 binding sequence in the binding and substrate-binding peptides mediates MMP-14 targeted retention of these probes, while the substrate peptide without the binding sequence could potentially diffuse away from target areas even after the NIRF signal is specifically activated by MMP-14. These peptide probes could also pool in necrotic GBM regions, similar to other small molecule radiographic contrast agents and cyanine dye-containing peptides 39-43, which could aid the neurosurgeon's assessment of the tumor. Employing the cocktail mixture containing the [^64^Cu]Cu-binding peptide and non-labeled substrate peptide in these studies showed a direct correlation between the separate PET and NIRF signals within GBM orthotopic xenografts, which was similar to prior results using the single [^64^Cu]Cu-substrate-binding peptide probe for both PET and NIRF imaging in an alternative patient derived xenograft GBM model 29. As opposed to the NIRF signals observed throughout the orthotopic tumor for the non-labeled substrate peptide that yielded a favorable DSC value (0.73), the [^64^Cu]Cu-binding peptide showed lower volumetric overlap with the tumor as indicated by a relatively lower DSC value (0.45). Both the non-labeled and radiolabeled substrate-binding peptide showed favorable overlap between orthotopic tumors regions and the respective NIRF and PET signals (DSC >0.68). Overall, these results support both PET and NIRF imaging with the substrate-binding peptide, and NIRF imaging with the substrate peptide, for whole-tumor visualization of GBM orthotopic xenografts. Imaging probes that are retained in both viable and necrotic GBM regions is clinically desirable for surgical management of GBM, since neurosurgeons must identify and resect both regions where safely feasible for patient care.

The present quantitative DSC analyses of signal overlap with GBM xenografts built upon prior qualitative results that showed GBM-specific localization of NIRF or PET signals from the MMP-14 targeted peptide probes 29. In addition to physiological localization of the imaging agents, DSC values determined in this study may be affected by the accuracy of tissue mask registration from the semi-automatic methods used to re-size the 2-dimensional fluorescence and histology images. Potential errors in registration may occur when fusing 3-dimensional PET/CT and MRI-defined tissue regions and associated VOIs, although readily discernable anatomical landmarks (skull base, temporal-mandibular junction, etc.) were used for reference among all imaging time points. Several preclinical studies using other imaging probes have quantitatively evaluated spatial overlap between *in vivo* tumor and fluorescence contrast agents 37, 38, 44, 45 or PET imaging agents 46, 47 in GBM orthotopic models, although differences in methodology preclude direct comparisons with the DSC values for the MMP-14 targeted peptide probes in the present studies. Human studies using radiolabeled amino acid analogs or peptides for imaging brain tumors have demonstrated DSC values ranging from 0 to 0.71 between PET signals and MRI-defined tumor volume 36, 48-54. Factors that influence intra-GBM localization of imaging agents in both human and preclinical studies include heterogeneous expression of molecular targets, vascularization, cellular density, integrity of the blood brain barrier, and necrosis 36, 48-54.

The preclinical studies above provided direct comparison of the optical contrast from the MMP-14 targeted peptide probes and 5-ALA in human GBM orthotopic xenografts relative to normal brain in mice. Imaging at 4 h after administering the contrast agents was a suitable time point for detecting PpIX fluorescence from 5-ALA and activated NIRF signals from the MMP-14 targeted peptide probes, consistent with prior work using these contrast agents in mice bearing orthotopic xenografts 29, 34, 38, 55. The peptide probes provided comparable TBR and higher tumor-specific localization (higher DSC) relative to 5-ALA for detecting bulk GBM orthotopic xenografts in these studies. The notable amount of extra-tumor PpIX fluorescence contrast observed indicates relatively poor specificity of 5-ALA in the human GBM models used in our studies. Exploring alternative GBM models that more closely mimic clinical GBM could provide greater insights into the relative differences and similarities between 5-ALA and novel imaging probes to inform future translational research. Previous studies have shown PpIX fluorescence in normal brain regions (white matter tracts, hippocampus) as well as variable tumor-specific imaging contrast across different orthotopic xenograft models after administering 5-ALA to mice 34, 38, 44, 55. Clinical studies using 5-ALA in patients with malignant glioma have shown 75-96% sensitivity and 29-96% specificity for intraoperative detection of malignancy 11-13, 56, 57; varying specificity for 5-ALA across clinical studies has been explained by variable proportions of normal tissue specimens collected during surgery and by PpIX fluorescence observed in non-malignant pathologies (e.g., reactive astrocytes) 12, 14, 58. Recent cellular-level fluorescence analyses of human GBM tissues indicated that PpIX preferentially accumulates within myeloid cells associated with GBM and also accumulates in extracellular spaces 59.

Intraoperative detection of diffusely invading malignant cells for maximal safe resection remains a known clinical challenge in human patients with GBM. These regions demonstrate variable fluorescence contrast that impacts the overall accuracy of 5-ALA for intraoperative imaging during neurosurgery of GBM 60, 61. The U87 and D54 human GBM models used in the present studies showed a relatively defined boundary of tumor cells at the xenograft edge, which limits how accurately these models represent human GBM 62, 63. This factor precluded assessment of whether NIRF contrast from the peptides could identify diffusely spreading GBM cells beyond the bulk tumor. However, NIRF signal contrast from the substrate peptide was apparent in a brain region of a mouse harboring microscopic deposits of GBM cells (approximately 17% GBM cells relative to total number of cells in the H+E and MMP-14 immunofluorescence field of view) in the present studies (Figure 1D). These results warrant further investigations to evaluate the extent of diffusely invading GBM cells that can be detected using MMP-14 targeted imaging probes.

The present in-situ resection experiments indicated that both NIRF and PET imaging with the MMP-14 targeted peptide probes successfully detected residual tumor regions in the surgical cavity during GBM xenograft debulking. Real-time intraoperative NIRF imaging avoids potential challenges that would be associated with quantitative PET image-guided resection of GBM, such as brain shift upon opening the dura and partial volume effects in relatively small VOIs 64. Our findings support further assessment of the MMP-14 targeted peptide probes during image-guided resection of GBM, such as directly comparing the extent of NIRF-guided relative to 5-ALA-guided resection of preclinical GBM models as reported in other studies 63, 65. Significantly greater DSC for the NIRF signals from the peptides relative to PpIX fluorescence in the present studies suggests that the MMP-14 targeted peptide probes would provide superior intraoperative accuracy relative to 5-ALA in the human GBM orthotopic models used here. The extent of safe resection is the greatest factor considered by neurosurgeons when treating human patients with brain tumors; residual bulk or invading tumor growth cannot be removed if found in eloquent brain regions, regardless of the accuracy of the imaging probe used to aid visualization. Novel imaging probes would be of greatest benefit for identifying safely resectable brain regions harboring low-grade or infiltrating glioma cells, which are challenging to detect using currently approved imaging approaches. Future work is required to determine if the MMP-14 targeted peptide probes used in these studies meet such criteria.

## Conclusion

Non-inferior TBR and superior tumor-specific localization of the peptide probes compared to 5-ALA supports additional development of MMP-14 targeted imaging approaches to aid assessment of GBM. Qualitative and quantitative analyses indicated imaging signals from the MMP-14 targeted peptide probes localized in tumor regions during *in situ* resection. The collective results support the concept for image-guided surgery of GBM using MMP-14 targeted imaging probes.

## Supplementary Material

Supplementary materials and methods, figures.

## Figures and Tables

**Table 1:** *Ex vivo* mean fluorescence intensity (MFI) from the substrate peptide, substrate-binding peptide, or PpIX measured in 1 mm formalin-fixed brain slices from mice bearing U87 or D54 human GBM orthotopic xenograft tumors.

	MFI in bulk tumor	MFI in normal brain	p value (tumor vs. normal brain MFI)
Substrate peptide	0.013 ± 0.011	0.001 ± 0.001	<0.01
Substrate-binding peptide	0.008 ± 0.005	0.001 ± 0.001	<0.05
PpIX	598 ± 330	172 ± 182	<0.001

## Abbreviations

5-ALA: 5-aminolevulinic acid
ANOVA: analysis of variance
CT: computed tomography
FDA: Food and Drug Administration
FFPE: formalin fixed paraffin embedded
GBM: glioblastoma multiforme
H+E: hematoxylin and eosin
*i.v.*: intravenous
mAb: monoclonal antibody
MFI: mean fluorescence intensity
MMP: matrix metalloproteinase
MRI: magnetic resonance imaging
NIR: near-infrared
NIRF: near-infrared fluorescence
NOTA: 1,4,7-triazacyclononane-1,4,7-triacetic acid
PET: positron emission tomography
*p.i.*: post injection
PpIX: protoporphyrin IX
ROI: region of interest
SBR: signal-to-normal brain ratio
SUV_max_: maximum standardized uptake value
SUV_mean_: mean standardized uptake value
SUVR: standardized uptake value ratio
TBR: tumor-to-normal brain ratio
UAB: University of Alabama at Birmingham
VOI: volume of interest
