# Supplementary material for: Comparison of 5-aminolevulinic acid and MMP-14 targeted peptide probes in preclinical models of GBM
Source: Theranostics. 2025 Feb 24;15(8):3517–31. doi: 10.7150/thno.107210 (PMC11905129; doi:10.7150/thno.107210)
Supplement: Supplementary file 1 — Supplementary materials and methods, figures. [file thnov15p3517s1.pdf]

SUPPLEMENTARY MATERIAL

Supplementary Materials and Methods

General reagents

All general reagents were from commercial suppliers (Thermo Fisher, Waltham, MA, USA; Sigma, St. Louis, MO, USA) unless specified otherwise. 5-ALA hydrochloride (Sigma, A7793-1G) was dissolved at 25 mg/mL in sterile PBS and stored at -20 ºC prior to use. Primary antibody for MMP-14 (rabbit anti-MMP-14 monoclonal antibody (mAb), clone EP1264Y) was from Abcam (Cambridge, MA). Fluorophore-conjugated goat anti-rabbit polyclonal secondary antibody was from Invitrogen (Thermo Fisher). ^{64}Cu was obtained in 0.1 M HCl from the University of Alabama at Birmingham (UAB) Cyclotron Facility.

Immunofluorescence

Unstained FFPE tissue sections (5 µm) were deparaffinized and antigen was retrieved by heating for 10 min at 95 °C in citrate buffer pH 6 with 1 mM EDTA. Tissues were blocked in 5% bovine serum albumin (BSA)/TBST at room temperature, incubated overnight at 4 °C with anti-MMP-14 mAb (Abcam ab51074) at 1 µg/mL in 5% BSA/TBST, washed in TBST, incubated for 3 h at room temperature with AlexaFluor 546-goat anti-rabbit secondary antibody (A11035, Invitrogen, Thermo Fisher) at 1/1000 dilution in 5% BSA/TBST, rinsed in TBST, and mounted in DAPI-Fluoromount-G (Southern Biotech, Birmingham, AL). Slides were imaged on an EVOS M7000 scanner (Invitrogen, ThermoFisher) using the DAPI and RFP channels and the 4X objective. H+E stained slides from the same brain slice were imaged on the EVOS M7000 scanner using the brightfield channel and the 4X objective. Integrated instrument software was used to generate whole-tissue composites from multiple fields of view. Mean fluorescence intensity (MFI) values of the tumor area and contralateral normal brain were quantified using ImageJ by drawing manual regions of interest (ROIs) based on H+E stained tissue obtained from the same brain slice. Quantification of total cell number in fields of view of interest was performed using QuPath (v0.5.1) based on nuclei apparent in the DAPI channel after appropriate thresholding; manual ROIs outlining GBM cells (based on H+E and MMP-14 immunofluorescence) were generated in QuPath and used to determine the percent GBM cells relative to total number of cells in fields of view of interest.

Production of peptide probes and radio HPLC analyses

The MMP-14 binding, substrate, and substrate-binding peptide probes were produced by solid phase techniques as previously described (1); the [^{64}Cu]Cu-substrate-binding peptide (previously referred to as "^{64}Cu-substrate-binding peptide") was produced using previously described procedures (1). Radiolabeled [^{64}Cu]Cu-binding peptide was produced by reacting ^{64}Cu (43.5-76.6 MBq) in 0.5 M sodium acetate pH 5.9 with the binding peptide (0.21 mM) at room temperature. Radiolabeled [^{64}Cu]Cu-substrate-binding peptide was produced by reacting ^{64}Cu (53.6 MBq) in 0.5 M sodium acetate pH 5.9 containing acetonitrile (approximately 35% by volume) with the substrate-binding peptide (0.1 mM) at 37 ºC. Radiolabeling reactions were monitored by reversed-phase HPLC (RP-HPLC) as previously described (1). Briefly**,** RP-HPLC analyses were performed on an Agilent 200 liquid chromatography system outfitted with an Agilent Zorbax SB-C18 RP-HPLC column (3x250 mm, 5 µm particle size) and a matching guard cartridge (3x7 mm). Gradient elutions at a flow rate of 0.5 mL/min were performed with water containing 0.1% trifluoroacetic acid (solvent A) and acetonitrile (solvent B) according to the

following method: 0-15 min linear gradient from 90% A/10% B to 60% A/40% B, 15-17 min linear gradient to 5% A/95% B, 17-20 min hold at 5% A/95% B, 20 min return to 90% A/10% B and equilibrate. UV-Vis was monitored at 220, 278, and 775 nm. An attached radiodetector (model 105-S, Carroll & Ramsey Associates) was used to monitor elution of radioactive compounds.

Animal subjects and husbandry

Animal studies were approved by the University of Alabama at Birmingham Institutional Animal Care and Use Committee (20366) and performed in compliance with guidelines from the Public Health Service Policy and Animal Welfare Act of the United States. Mice were anesthetized with isoflurane during all dosing and live imaging procedures. Anesthetized mice were euthanized by cervical dislocation.

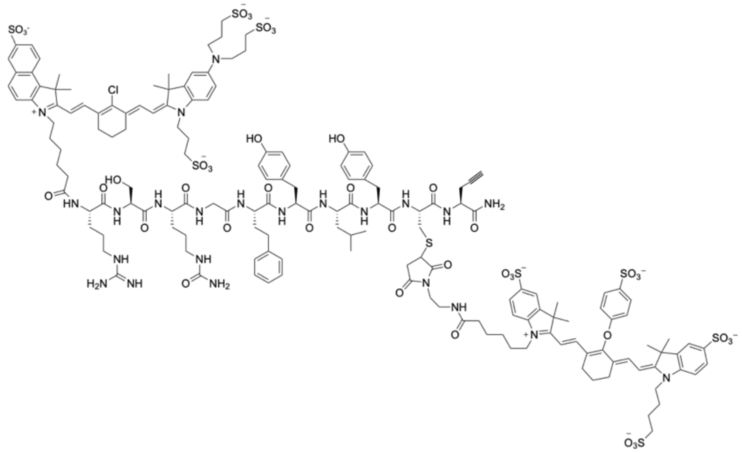

Figure S1. Structure of the substrate peptide.

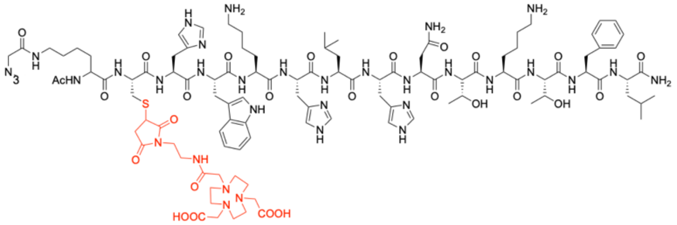

Figure S2. Structure of the binding peptide.

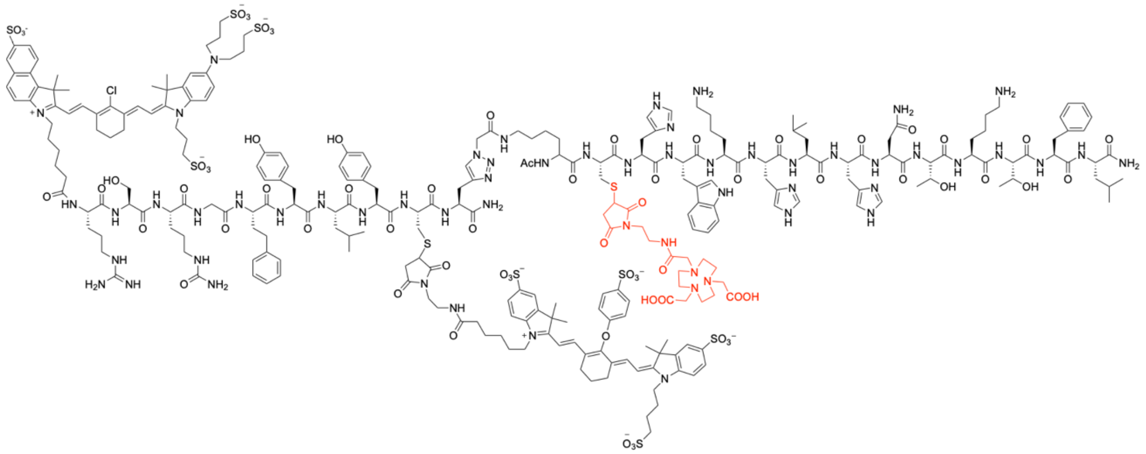

Figure S3. Structure of the substrate-binding peptide.

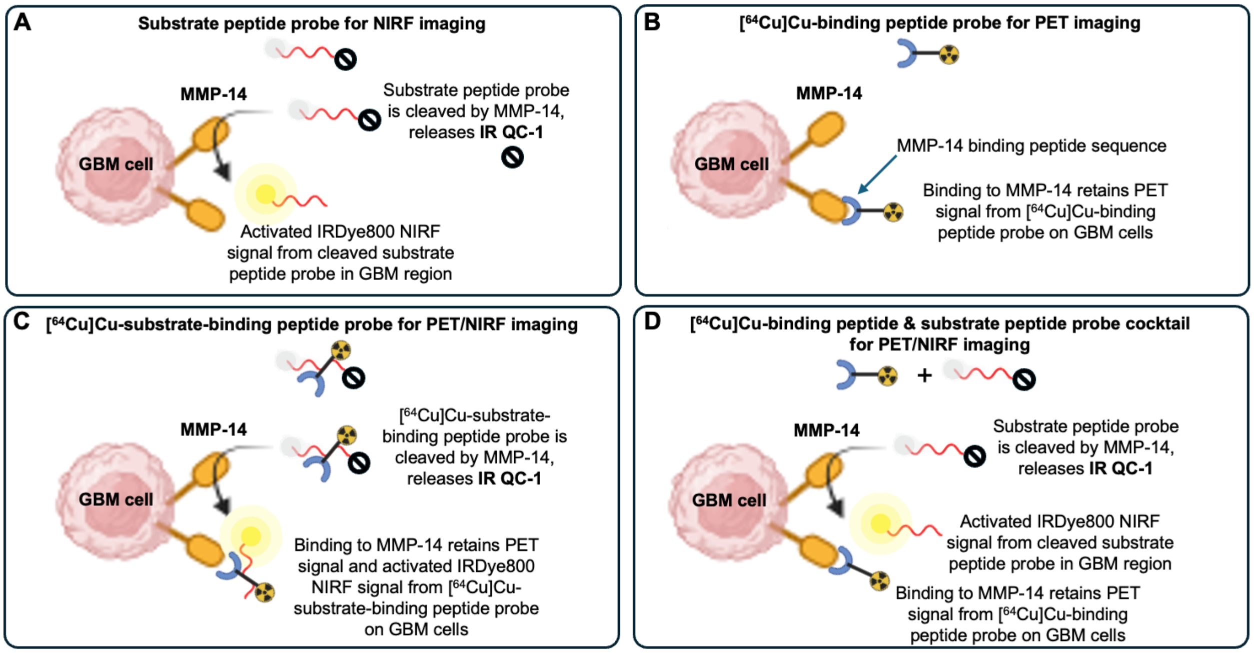

Figure S4. Diagram showing the molecular activity of the MMP-14 targeted peptide probes interacting with MMP-14 expressed on GBM cells for single and dual-modality NIRF and PET imaging of GBM. A, substrate peptide (NIRF imaging probe). B, [^{64}Cu]Cu-binding peptide (PET imaging probe). C, [^{64}Cu]Cu-substrate-binding peptide (single probe for dual NIRF and PET imaging). D, cocktail mixture containing the substrate peptide and the [^{64}Cu]Cu-binding peptide probes (dual NIRF and PET imaging). (Generated with BioRender.com)

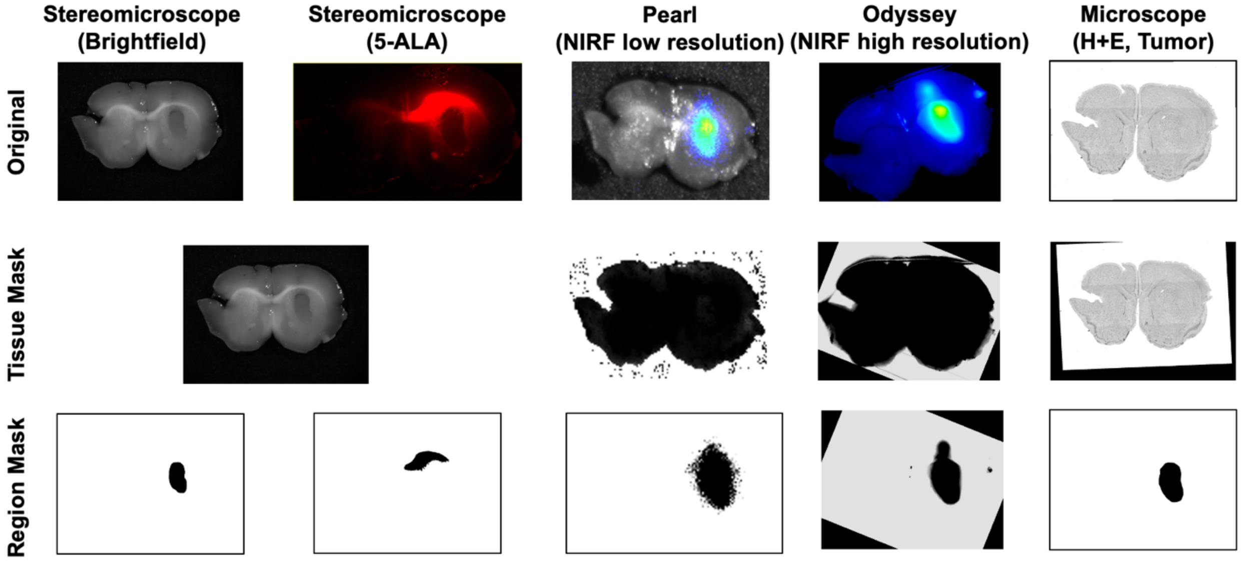

Figure S5. Analysis workflow diagram for 2-dimensional images of ex vivo fixed brain tissue from mice bearing GBM orthotopic xenografts; a representative example is provided from a U87 xenograft-bearing mouse administered the substrate peptide and 5-ALA.

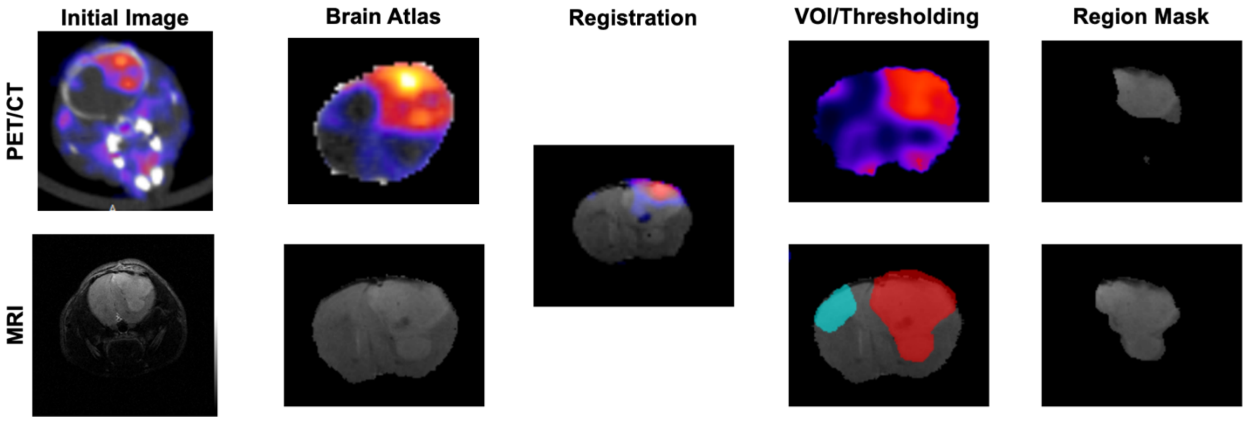

Figure S6. Analysis workflow diagram for 3-dimensional images (MRI, PET/CT) of in vivo tissue regions from mice bearing GBM orthotopic xenografts; a representative example is provided from a U87 xenograft-bearing mouse administered the [^{64}Cu]Cu-substrate-binding peptide.

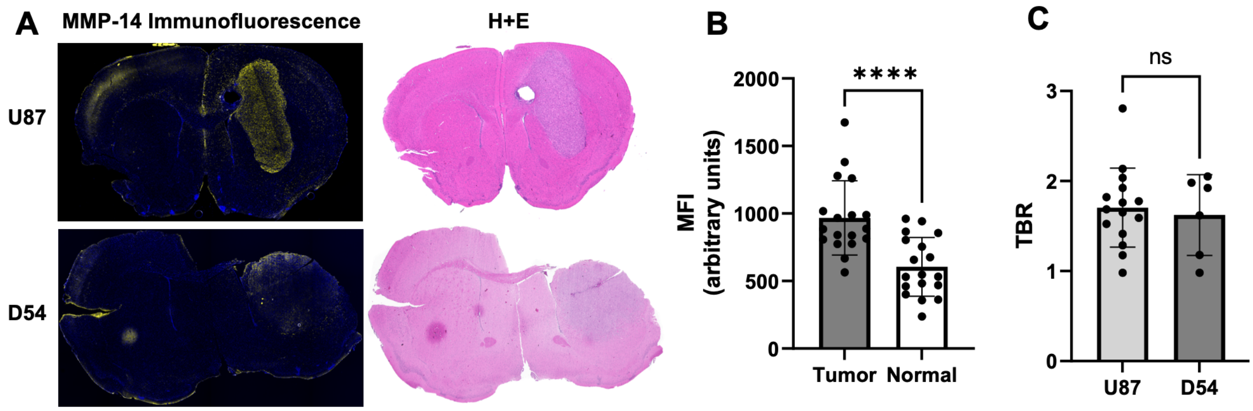

Figure S7. A, representative MMP-14 immunofluorescence (yellow; nuclei counterstained with DAPI, blue) and H+E stained brain tissue sections from mice bearing U87 (upper panels) or D54 (lower panels) GBM orthotopic xenografts. B, MFI (mean±SD) of MMP-14 immunofluorescence in tumor area and contralateral normal brain regions of tissue sections from mice bearing U87 or D54 GBM orthotopic xenografts; significance determined by paired *t*-test (****, $p<0.0001$). C, tumor-to-normal brain ratio (TBR, mean±SD) of MMP-14 immunofluorescence in brain tissue sections according to tumor type; significance determined by Mann-Whitney test (ns, not significant).

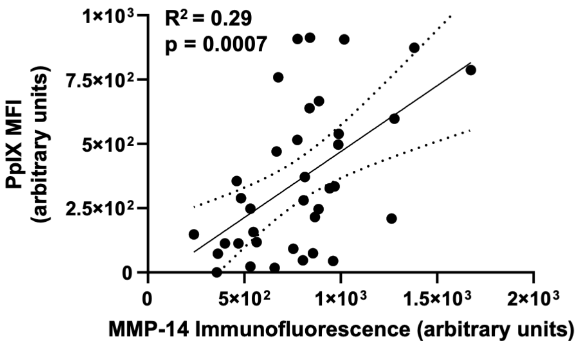

Figure S8. Least-squares fit of linear regression between histological MMP-14 immunofluorescence MFI and PpIX MFI in 1 mm brain slices from nude mice bearing U87 or D54 GBM orthotopic xenografts; dotted lines show the 95% confidence interval of the correlation.

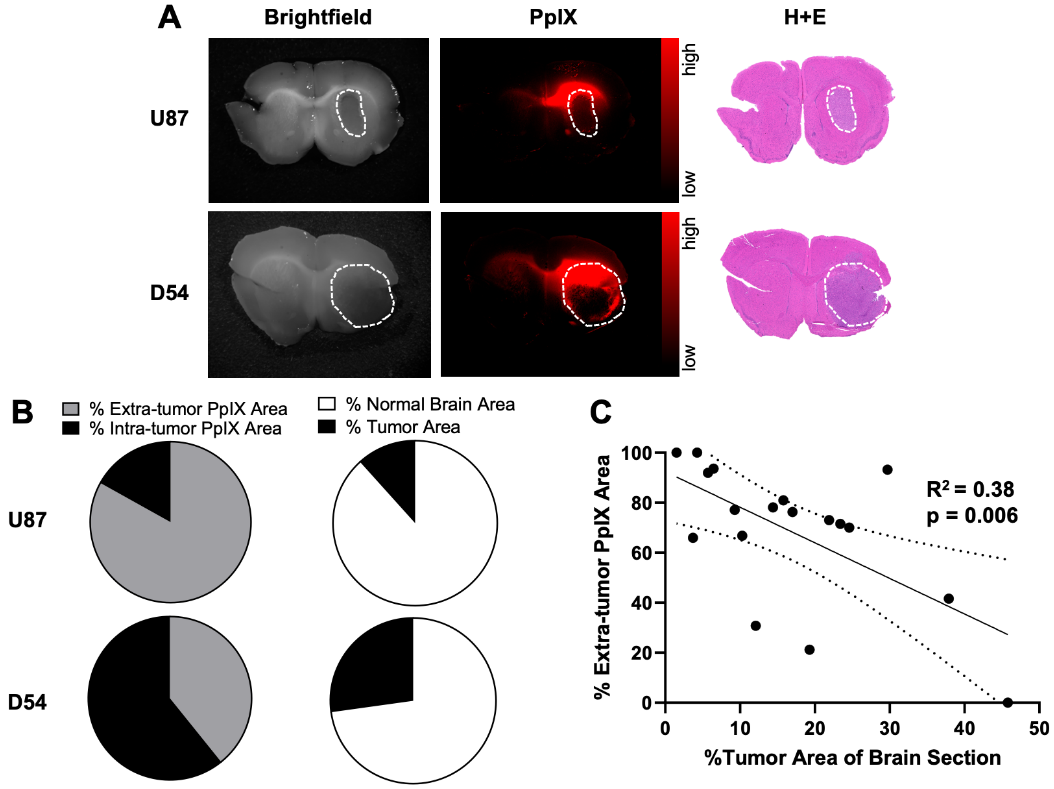

Figure S9. A, representative images of fixed brain slices from mice bearing U87 (top panels) or D54 (bottom panels) GBM orthotopic xenografts; corresponding brightfield and PpIX fluorescence panels are from the same 1 mm brain slice, which was also used to obtain a histologic section for the corresponding H+E panel. White dotted line indicates tumor region. B, pie charts indicating mean percent intra-tumor and extra-tumor PpIX fluorescence area relative to the whole PpIX fluorescence-positive area of the brain section (left panels), or the mean percent area of tumor or normal brain regions relative to the whole brain section area (right panels) of fixed brain slices from mice bearing U87 (top panels) or D54 (bottom panels) GBM orthotopic xenografts. C, least-squares fit of linear regression between percent area of tumor relative to the whole brain section area and the percent extra-tumor PpIX fluorescence area relative to the whole PpIX fluorescence-positive area of the brain section; dotted lines show the 95% confidence interval of the correlation.

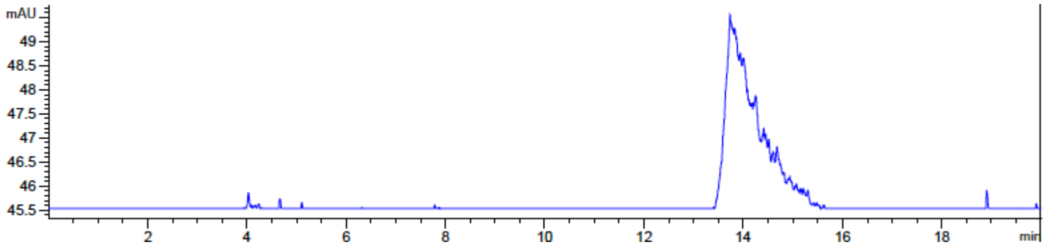

Figure S10. Radio RP-HPLC analysis of [^{64}Cu]Cu-binding peptide reaction. Product retention time: 13.7 min. Retention time of the non-labeled binding peptide: 13.4 min.

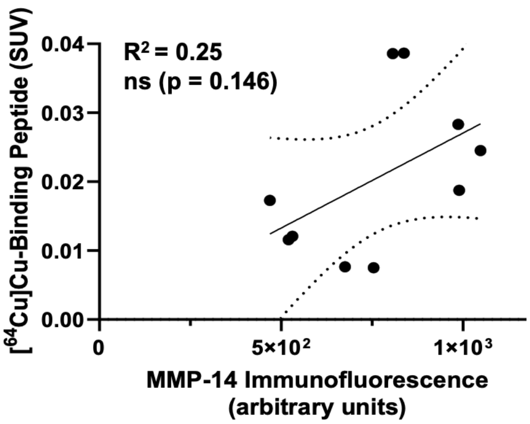

Figure S11. Least-squares fit of linear regression between ex vivo 2-dimensional histological MMP-14 immunofluorescence MFI and in vivo 3-dimensional PET (SUV_{mean}) signals from [^{64}Cu]Cu-binding peptide in nude mice bearing U87 or D54 GBM orthotopic xenografts; dotted lines show the 95% confidence interval of the correlation.

References

1. Kasten BB, Jiang K, Cole D, Jani A, Udayakumar N, Gillespie GY, et al. Targeting MMP-14 for dual PET and fluorescence imaging of glioma in preclinical models. European Journal of Nuclear Medicine and Molecular Imaging. 2020;47(6):1412-26.
